# Re-thinking global health sector efforts for HIV and tuberculosis epidemic control: promoting integration of programme activities within a strengthened health system

**DOI:** 10.1186/1471-2458-10-394

**Published:** 2010-07-05

**Authors:** Dermot Maher

**Affiliations:** 1 Medical Research Council (MRC)/Uganda Virus Research Institute (UVRI) Uganda Research Unit on AIDS, PO Box 49 Entebbe, Uganda

## Abstract

**Background:**

The global financial crisis threatens global health, particularly exacerbating diseases of inequality, e.g. HIV/AIDS, and diseases of poverty, e.g. tuberculosis. The aim of this paper is to reconsider established practices and policies for HIV and tuberculosis epidemic control, aiming at delivering better results and value for money. This may be achieved by promoting greater integration of HIV and tuberculosis control programme activities within a strengthened health system.

**Discussion:**

HIV and tuberculosis share many similarities in terms of their disease burden and the recommended stratagems for their control. HIV and tuberculosis programmes implement similar sorts of control activities, e.g. case finding and treatment, which depend for success on generic health system issues, including vital registration, drug procurement and supply, laboratory network, human resources, and financing. However, the current health system approach to HIV and tuberculosis control often involves separate specialised services. Despite some recent progress, collaboration between the programmes remains inadequate, progress in obtaining synergies has been slow, and results remain far below those needed to achieve universal access to key interventions. A fundamental re-think of the current strategic approach involves promoting integrated delivery of HIV and tuberculosis programme activities as part of strengthened general health services: epidemiological surveillance, programme monitoring and evaluation, community awareness of health-seeking behavior, risk behaviour modification, infection control, treatment scale-up (first-line treatment regimens), drug-resistance surveillance, containing and countering drug-resistance (second-line treatment regimens), research and development, global advocacy and global partnership. Health agencies should review policies and progress in HIV and tuberculosis epidemic control, learn mutual lessons for policy development and scaling up interventions, and identify ways of joint planning and joint funding of integrated delivery as part of strengthened health systems.

**Summary:**

As both a danger and an opportunity, the global financial crisis may entail disaster or recovery for global health sector efforts for HIV and tuberculosis epidemic control. Review of policies and progress in control paves the way for identification of synergies between the two programmes, within strengthened health services. The silver lining in the global economic crisis could be better control of the HIV and tuberculosis epidemics, better overall health system performance and outcomes, and better value for money.

## Background

The silver lining in the cloud of the global economic crisis is the enforced fundamental re-think of the status quo, not only in the financial world, but also in many other spheres affected by the crisis. "The crisis is too good to waste" - this means for global health it is opportune to reconsider established practices and policies with the aim of improving health system performance and delivering better results and value for money. This will not only help during the economic downturn to maintain the momentum of recent international health gains but will also prepare the ground for even greater health gains when the global economy recovers. The global financial crisis threatens global health, particularly exacerbating diseases of inequality such as HIV/AIDS and diseases of poverty such as tuberculosis. The approaches to HIV and tuberculosis epidemic control are ripe for a re-think. In this paper we look for a possible silver lining in the global economic crisis regarding health sector efforts for the control of HIV and tuberculosis, which are both among the leading infectious causes of illness and death worldwide [1].

HIV and tuberculosis share many similarities in epidemic characteristics and challenges in mounting an effective health sector response [2]. Despite also considerable overlap in epidemiology [2], the status quo is that not enough has been made of the opportunities for mutual learning and interaction between the respective control programmes. Re-thinking the status quo can help both programmes in two ways: firstly, to learn from each other's experiences with the aim of expediting implementation of their respective stratagems for epidemic control, and secondly, to improve collaboration and advance progress in areas of mutual concern. As part of efforts to strengthen overall health system performance, maximizing synergies and decreasing inefficiencies in the approaches to HIV and tuberculosis epidemic control can generate substantial health gains.

The aim of this paper is to re-think established practices and policies for HIV and tuberculosis epidemic control. First there is a comparison between HIV and tuberculosis, showing the important differences, similarities and overlap between these epidemics. This then enables identification of the similarities in the main stratagems for HIV and tuberculosis epidemic control. Review of the current status of the HIV and tuberculosis epidemics and of implementation of control measures leads to the conclusion that these epidemics are far from under control, necessitating a re-think of established practices and policies. Based on the similarities between control stratagems, approaches are proposed for better HIV and tuberculosis epidemic control. The opportunities identified for greater synergies between these programmes and the health system could deliver better results and value for money. Although the main focus is on developing countries, which bear the brunt of both epidemics and have the least resources for the health sector response, lessons may also be applicable to developed countries.

## Discussion

### HIV and tuberculosis - differences, similarities and overlap

HIV is very new and infection is as yet incurable; tuberculosis very old and has been curable with drugs for the past 60 years. The HIV epidemic arose in the twentieth century following the cross-species transfer of simian immunodeficiency viruses from primates to humans [3]. HIV currently infects 33 million out of the global population of 6 billion people, i.e. 0.5% of the world's population [4]. HIV infection is lifelong, and fatal if untreated. The virulence of HIV as a new human pathogen mitigates against its success and its further spread may depend on its evolution with selection of less virulent strains. In contrast to the modernity of HIV, the *Mycobacterium tuberculosis *complex clonal group responsible for causing tuberculosis has co-evolved with humanity over the past three million years - our remote early hominid ancestors may have suffered from tuberculosis [5]. Once infected with *M. tuberculosis*, a person remains infected for many years, probably for life. The vast majority (90%) of people who are infected with *M. tuberculosis *do not develop tuberculosis [6]. The risk of tuberculosis following *M. tuberculosis *infection is determined mainly by the individual's immune status (and hence HIV infection is a potent risk factor for tuberculosis). Even in the absence of treatment, self-cure occurs in three out of ten patients with tuberculosis (without HIV infection) [7]. The success of *M. tuberculosis *as a human pathogen, infecting about one-third of the world's population, reflects the extremely long and close relationship it has enjoyed with humanity [8].

The nature of the interaction between the two human pathogens, with a close relationship between HIV and tuberculosis, is a consequence of the fact that HIV is a very new and *M. tuberculosis *a very old pathogen. HIV and *M. tuberculosis *share several similarities in their interaction with the human host. They are both life-long infections, with a long and variable latent period between infection and onset of disease - in the case of HIV, about two years in Africa [9] (longer in developed countries) and in the case of *M. tuberculosis*, usually less than five years [10]. In both cases, the outlook for the infected individual is worse the later the detection of disease and the start of treatment [11,12]. The annual global toll of deaths from HIV and from tuberculosis is similar - in 2007 there were 1.5 million deaths from HIV [4] (excluding those with tuberculosis), 1.3 million deaths from tuberculosis (among HIV-negative people) [13], and 0.5 million deaths among people with HIV and tuberculosis [13]. The worldwide epidemics of HIV and tuberculosis are both out of control, with HIV still infecting people faster than the pace of antiretroviral treatment roll-out [4] and the absolute number of annual tuberculosis cases still increasing [13].

There is a considerable overlap between those infected with HIV and those infected with *M. tuberculosis*, especially in sub-Saharan Africa [14], with tuberculosis a leading cause of death among people with HIV infection [14]. Tuberculosis and HIV control programmes clearly have mutual concerns: the prevention of HIV infection and the treatment of HIV/AIDS should be components of tuberculosis control, and tuberculosis care and prevention should be priorities in the management of HIV/AIDS [15,16]. Despite recognition of the potential benefits of collaboration between tuberculosis programmes and HIV/AIDS programmes they have often continued to pursue separate courses [17]. They can no longer afford to do so in an era of global economic crisis which demands maximum cost efficiencies. The exigencies of the global economic crisis provide an impetus for HIV programmes and tuberculosis programmes together to take stock of their activities and progress in epidemic control. They need to consider not only how to improve collaboration aimed at advancing progress in the areas of mutual concern, but also how to learn from each other's experiences with the aim of advancing progress in implementing their respective main stratagems for epidemic control.

### Stratagems for HIV and tuberculosis epidemic control

The principle of infectious disease epidemic control is to reduce the average number of people infected by each infectious case so that the case reproduction number is less than one. This results in declining incidence of infection. The main stratagems for HIV and tuberculosis epidemic control include: prevention of primary infection, modification of risk factors for infection, drug prophylaxis, decreased transmission by treatment of infected individuals (treatment as prevention), and vaccination to prevent progression from infection to disease. The emphasis has been on different stratagems for HIV and for tuberculosis epidemic control, with decreased transmission by treatment of infected individuals (treatment as prevention) gaining attention recently for HIV, whereas this has been the mainstay of tuberculosis control for the past 60 years.

The emphasis in HIV control has until recently mainly been on promoting behavioural modifications aimed at decreasing the risk of primary infection [18]. In revolutionising the care of people with HIV, antiretroviral therapy (ART) has opened the door to treatment as prevention. The benefits of early ART initiation are not only improved individual patient outcomes but also reduced infectiousness and therefore decreased HIV transmission [19]. How best to use ART for prevention has emerged as the most pressing question that faces HIV/AIDS science [20]. Could universal voluntary testing with immediate ART be a strategy for elimination of HIV transmission? A mathematical model shows that this strategy could greatly accelerate the transition from the present endemic phase, in which most adults with HIV infection are not on ART, to an elimination phase, in which most are on ART, within 5 years [21]. There is an urgent need for research to establish the feasibility of this approach and validate the modeling results.

Removal of tuberculosis patients from their homes for isolation in sanatoria may have played a role in decreasing *M. tuberculosis *transmission in the pre-chemotherapy era. However the development of specific anti-tuberculosis chemotherapy 60 years ago raised the prospect of reducing transmission risk through decreased infectiousness of the index cases [22]. This can be achieved by prompt diagnosis and effective treatment, which lie at the heart of modern approaches to tuberculosis control [23]. With proper treatment, a person with infectious tuberculosis very quickly becomes non-infectious - probably most often in less than two weeks - and so can no longer transmit infection to others [24].

Shortening the period of infectivity can maximize the impact of ART and of antituberculosis chemotherapy on transmission of HIV and *M. tuberculosis *respectively. In the case of HIV, substantial transmission occurs while the infected person is asymptomatic, so shortening the period of infectivity may well require regular testing of asymptomatic people for HIV infection, and testing of people after an at-risk exposure, as well as prompt diagnosis and effective treatment of people when they present with symptoms of HIV-related disease. In the case of tuberculosis, transmission occurs from patients with pulmonary tuberculosis when they are symptomatic with cough, so early diagnosis requires patient access to quality health services providing rapid and reliable diagnosis. In both cases, promotion of community awareness is necessary for effective health-seeking behaviour, so that people understand and accept the need for testing as early as possible to maximize the possibility of effective action to decrease transmission.

The specific interventions to address HIV and tuberculosis can be grouped under the main stratagems for epidemic control (prevention of primary infection, modification of risk factors for infection, drug prophylaxis, decreased transmission by treatment of infected individuals, and vaccination to prevent progression from infection to disease) (Table 1) [25-45].

**Table 1:** Specific interventions to address HIV and tuberculosis grouped under the main stratagems for epidemic control

Stratagem	HIV	Tuberculosis
*Prevention of primary infection*		

behavioural modification	decreased risk exposure, e.g. safe sex and decreased sex partners [25] and safe injecting drug use [26]	education, e.g. cough hygiene [27]

environmental modification	decreased risk of occupational exposure through safe handling and disposal of sharps	environmental measures to decrease nosocomial transmission [27,28] (particularly important where HIV prevalence is high)

protect site of infection	vaginal microbicides (under evaluation) [29]	face masks (NN95 specification) [27]

*Modification of risk factors for infection*		

promote decreased substance abuse	decreased alcohol [30] and drugs [30]	decreased alcohol [31] and smoking [32]

detect and treat conditions associated with increased risk	treatment of sexually transmitted infections [33]	detection and management of diabetes [34]

modify personal biological characteristic	male circumcision [35]	

*Drug prophylaxis*		

pre-exposure	pre-exposure prophylaxis (under evaluation) [36]	isoniazid preventive treatment for infants born to mothers with tuberculosis [37]

pre- and post-exposure	prevention of mother to child transmission [38]	

post-exposure	post-exposure prophylaxis [39]	isoniazid preventive treatment for people with latent *M. tuberculosis *infection or for people at high risk of recurrent tuberculosis [40]

*Decreased transmission by treatment of infected individuals (treatment as prevention) *[41]		

	prompt diagnosis and effective treatment of people with symptomatic HIV-related disease [42]	prompt diagnosis and effective treatment of people with symptomatic pulmonary disease [23]

	proposal for prompt diagnosis among asymptomatic individuals either through an individual seeking a test for HIV after an at-risk exposure or through regular universal testing [43]	efforts aimed at decreasing diagnostic delay through community education, improved access to care, and improved quality of clinical care [44]

*Vaccination to prevent progression from infection to disease*		

	no vaccine yet available	Bacille Calmette-Guerin (BCG) vaccination [45]

### Current status of HIV and tuberculosis epidemics and of control measures

Assessment of the current status of the HIV and tuberculosis epidemics indicates that these epidemics are far from under control (Table 2) [2,4,13].

**Table 2:** Estimates of key selected indicators of current status of HIV and tuberculosis epidemics

Indicator	HIV	Tuberculosis
annual incident cases (all)	2·5 million incident infections in 2007 [4]	9.3 million incident cases in 2007 [13]

annual deaths	2.1 million [2] (includes 0.5 million people with tuberculosis and HIV co-infection) [13]	1.3 million (excludes 0.5 million people co-infected with HIV) [13]

annual incident cases of drug-resistant strains	Global estimate not available.	0.5 million in 2007 [13]

Although there has been considerable progress in global implementation of measures for HIV and tuberculosis epidemic control, there is still a long way to go before achieving universal access for all to HIV and tuberculosis diagnosis and treatment (Table 3) [13,46-50].

**Table 3:** Key selected indicators of progress in global implementation of measures for HIV and tuberculosis epidemic control

Measure	HIV	Tuberculosis
diagnosis (proportion diagnosed among all with HIV or tuberculosis)	No global figure available in UNAIDS report [46]. Based on data from 12 low and middle-income countries, 20% of people with HIV infection know their status [46].	5.5 million cases diagnosed and treated in 2007 under programmes in line with global strategy to Stop TB/9.3 million estimated incident cases (59%) [13].
	
provision of first-line treatment (proportion receiving treatment among all those needing it)	ART 4 million/9.5 million at the end of 2007 [46] (42%)	

treatment success rate	No global documentation of overall rate of successful treatment outcome ("highly heterogeneous monitoring systems and the use of non-standardised definitions across programmes create additional hurdles for accurately measuring the success of programmes") [46]	85% global treatment success rate in 2006 for patients with sputum smear-positive pulmonary tuberculosis treated in line with the global strategy to Stop TB [13].
	
	Average retention in ART treatment programmes in sub-Saharan Africa was 75% after one year and 62% after two years [47].	75% treatment success rate (patients with sputum smear-positive pulmonary tuberculosis) in the WHO Africa region in 2006 [13].

drug-resistance surveillance	25 countries "were planning or implementing" WHO's global strategy for prevention and assessment of HIV drug resistance [48]. Seven countries have reported results [48].	Data from 90,726 patients in 83 countries and territories between 2002 and 2007 [49].

diagnosis of drug-resistant cases	No global figure available from UNAIDS report [46]. In nine countries reporting results from surveillance of transmitted HIV drug resistance from areas where ART was first used in the country, the prevalence of transmitted resistance was less than 5% [46].	30,000 cases of multidrug-resistance among people with smear-positive pulmonary tuberculosis diagnosed in 2007/353,000 estimated total worldwide (8.5%) [13].

provision of second-line treatment (proportion receiving treatment among all those needing it)	Results of a survey by national HIV programmes in 41 countries showed that 3% of people receiving ART were on second-line regimens [50]. The estimated number of people needing second-line ART is unknown.	3,681 cases of multidrug-resistance among people with smear-positive pulmonary tuberculosis known to be treated in 2007 according to international guidelines/353,000 estimated total worldwide cases (1%) [13].

treatment success rate (among people treated for drug-resistant HIV)	No global figure available from UNAIDS report [46].	Although "the size of most country cohorts in 2004 was too small to allow any useful analysis", treatment success rate ranged between about 50-70% for cohorts of patients treated according to international guidelines [13].

Regarding activities requiring collaboration between HIV and tuberculosis programmes ("collaborative TB/HIV interventions"), despite some progress the implementation of these interventions is far below the need [46], and far below the targets for 2015 set out in the "Global Plan to Stop TB" (Table 4) [51].

**Table 4:** Indicators of progress in implementation of collaborative TB/HIV interventions

intervention	number of people accessing intervention in 2007
screening for tuberculosis among people with HIV	300,000/13 million target in Africa (2.3%) [13]

testing for HIV among notified tuberculosis cases	500,000/900,000 target in Africa (55.6%) [13]

provision of isoniazid preventive therapy to people living with HIV	27,000/33 million people living with HIV worldwide (0.1%) [46]

TB infection control in health and other congregate facilities	no quantitative measure ("progress in implementing infection control interventions has been very slow" [46])

### Re-thinking established practices and policies for effective epidemic control

A key question arises from consideration of the current status of the HIV and tuberculosis epidemics and of implementation of epidemic control measures: what is the most effective and efficient health system approach to ensuring more widespread and sustainable access to interventions for HIV and for tuberculosis epidemic control? One approach is to continue with more of the same, i.e. specialised services for HIV and for tuberculosis with what may in some ways be regarded as a third programme of TB/HIV collaboration. Alternatively, a fundamental re-think of the current strategic approach involves promoting integrated services for HIV and tuberculosis which are part of strengthened general health services.

The contrasting experiences of global HIV and tuberculosis control provide some insights in answering this question. Accompanying the development of antituberculosis chemotherapy regimens in the 1960s and 1970s, the move away from the previous policy of specialised tuberculosis diagnosis and treatment services to integration into primary care in most countries, played a critical role in facilitating widespread access [23]. After integrating tuberculosis services with general health services, and decentralizing with community support [52], the bottleneck in expanding access to tuberculosis diagnosis and treatment is the quality of the general health services. This was recognised in the early years of this decade by the second ad hoc committee on the tuberculosis epidemic: "progress in tuberculosis control... depends on actions which are beyond the specifics of tuberculosis control" [53]. Although the committee's recommendations to stakeholders in tuberculosis control included to "strengthen health systems, particularly primary care delivery", progress in harnessing tuberculosis control efforts to the cause of health system strengthening has been slow and the health system bottleneck remains.

Regarding HIV programmes, the process of ensuring widespread and sustainable access has largely been through specialised services, rather than decentralized, integrated services. The picture of service provision for HIV in many developing countries is a mix of specialised services and integrated services, but the process of integration is generally at an earlier stage than with tuberculosis programmes. The UNAIDS policy advice that "greater attention must be paid to integrating HIV services into primary health care as part of managing chronic diseases" [46] is yet to be widely translated into action.

Given the different stages of development of integrated services for HIV and for tuberculosis programmes, the picture for areas of collaborative activity between HIV and tuberculosis programmes is predictably complicated. Although in the early years of this decade, the World Health Organization developed a global policy on HIV and tuberculosis programme collaboration representing an integrationist approach, with HIV and tuberculosis programme activities integrated into general health services [54], this was soon replaced by a revised approach, with emphasis on collaboration between HIV and tuberculosis programmes and little reference to the need for integration with general health services [55]. This led to the establishment of what may be considered a third vertical programme on "TB/HIV" [56] alongside the existing HIV and tuberculosis programmes. The slow progress in expanding access to interventions of mutual concern to HIV and tuberculosis programmes may be attributable at least in part to the policy failure to promote integrated services (taking into consideration the need for attention to infection control) within strengthened general health services.

Many of the types of activities for epidemic control undertaken by HIV and tuberculosis programmes are the same but are often undertaken separately e.g. epidemiological surveillance, programme monitoring and evaluation, community awareness of health-seeking behavior, risk behaviour modification, infection control, treatment scale-up (first-line treatment regimens), drug-resistance surveillance, containing and countering drug-resistance (second-line treatment regimens), research and development for new diagnostics, drugs and vaccines, global advocacy and global partnership. Implementation of these activities by the two programmes depends on the same health system issues, including vital registration, drug procurement and supply, laboratory network, human resources, financing, and health sector reform. Identifying such activities where joint efforts for HIV and for tuberculosis epidemic control can strengthen health services would help to maximize synergies (Table 5) [4,11-13,27,44,46,49,51,52,57,58]. The global economic crisis highlights the urgent need for international and national health organizations to review systematically the activities of HIV and tuberculosis programmes to identify opportunities for greater synergies between these individual health programmes and the health system and to deliver better results and value for money.

**Table 5:** Examples of programme activities where joint efforts for HIV and for tuberculosis epidemic control can be better integrated into a strengthened health system

Programme activity	Challenge	Example of HIV and tuberculosis programme collaboration	Consequence for strengthened health system
Programme monitoring and evaluation	Difficulty in measurement of the success of HIV treatment programmes because of highly heterogeneous monitoring systems and use of non-standardised definitions across programmes [46]	Joint contribution to development of standard measures for monitoring success of treatment, e.g. ART for HIV infection, learning from experience of standard outcomes in global tuberculosis control [44]	Better global documentation of overall rate of successful outcome of treatment of HIV/AIDS a priority disease of poverty

Programme monitoring and evaluation	Lack of a vital registration system in many of the countries most badly affected by HIV or tuberculosis (only five countries in Africa have vital registration systems covering more than 25% of the population) [57]	Joint support of efforts to develop national vital registration systems	Improvements in coverage and quality of vital registration systems would be of considerable benefit for better data on deaths of people with priority diseases of poverty, e.g. HIV and tuberculosis

Infection control in health and other congregate facilities	Lack of quantitative measure of implementation of measures for HIV infection control and slow progress in implementing TB infection control interventions [46]	Joint contribution to development and implementation of effective health system policies for infection control in health and other congregate facilities	Strengthened ability of health system to protect patients from nosocomial infection

Raising community awareness of health-seeking behavior	Late presentation during disease progression of patients with HIV [11] and patients with tuberculosis [12]	Joint development and implementation of comprehensive communication measures aimed at raising community awareness of the importance of seeking health care earlier in the course of progression of priority diseases, e.g. HIV and tuberculosis	Better outcomes of treatment of patients presenting earlier in the course of disease, with health system efficiency savings

Risk behavior modification	High continued levels of behaviour involving personal risk, e.g. unsafe sex [4] as a risk for HIV infection, and lack of cough hygiene as a risk for transmission of tuberculosis [27]	Joint contribution to comprehensive health education aimed at promoting healthy behavior and decreasing risk of HIV and tuberculosis	Improved health system approach to behavior modification regarding risk of HIV and tuberculosis among a wide range of diseases

Treatment scale-up (first-line treatment regimens)	Inadequate access to effective treatment of HIV infection [46] and tuberculosis [13]	Joint contribution to development and implementation of health system policies for decentralized provision of treatment of priority diseases, e.g. ART for HIV/AIDS, based on experiences of decentralised treatment of tuberculosis [52]	Faster progress towards goal of universal access to key interventions for control of priority diseases of poverty

Drug-resistance surveillance	Despite recent progress, insufficient laboratory capacity in countries most badly affected by HIV and by tuberculosis for surveillance of resistance to antiretroviral [46] and antituberculosis [13] drugs	Joint support of development of national and international capacity for drug-resistance surveillance, including resistance to antiretroviral and antituberculosis drugs	Improved health system capacity for drug-resistance surveillance, including resistance to antiretroviral and antituberculosis drugs

Containing and countering drug-resistance (including rational use of second-line treatment regimens)	Failure to contain the spread of drug-resistance [46,49]	Supporting health system capacity to contain drug-resistance, by learning mutual lessons from experiences in HIV and tuberculosis treatment	Improved health system capacity to contain resistance to drugs used in treatment of a wide range of diseases

Research and development for new diagnostics, drugs and vaccines	Inadequate development of new diagnostics, drugs and vaccines, especially for tuberculosis [51]	Joint support of advocacy for increased funding, and for development of platforms, for research and development	More efficient and effective health system contribution to development of new technologies for disease control

Global advocacy for resources	Competition between advocates promoting resource mobilisation for different diseases	Joint advocacy for funding of strong health systems which are able to respond effectively across a range of disease priorities and benefit from synergies of approaches to different diseases	More effective health system based on funding of disease control commensurate with the burden of disease

Global partnership	Failure to maximize synergies and avoid duplication and dispersion of effort among partners	Joint support of global partnerships which embrace joint HIV and tuberculosis issues (e.g. Global Fund for AIDS, Tuberculosis and Malaria) and of more cohesive global HIV partnerships, learning from lessons of the global Stop TB Partnership [58]	More effective and efficient roles played by partners in global health partnerships and in global HIV and tuberculosis partnerships

There are recent signs of an increasingly favourable global policy environment for this approach. Last year WHO launched the effort to "Maximize positive synergies between global health initiatives and health systems" [59]. Dr Carissa Etienne, assistant Director-General, WHO, has commented that "The financial crisis poses some fundamental questions about the way the international community uses its resources. And the response is that while we clearly need more funds for health, we also need to identify opportunities to deliver better results and value for money...promoting greater synergies between health systems and individual health programmes are key to making this happen" [60]. Suggested reforms in international health financing include incorporating the Global Fund to Fight AIDS, Tuberculosis and Malaria and the Global Alliance on Vaccines and Immunisations in a global fund for all the health Millennium Development Goals, with a mandate to address priority health problems and key bottlenecks in health systems [61].

### Conclusion

The global financial crisis threatens global health [62], in particular exacerbating diseases of inequality such as HIV/AIDS [63] and diseases of poverty such as tuberculosis [64]. The benefits of collaboration between those concerned with HIV and tuberculosis epidemic control have been pointed out for a long time [65]. However, despite some recent progress, collaboration remains inadequate, progress in obtaining synergies has been slow, and results remain far below those needed to achieve the goal of universal access to key interventions. "Crisis" is a medical metaphor derived from the concept which probably predates Hippocrates of the turning point of a disease - the moment after which a patient either recovered or died. As both a danger and an opportunity, the global financial crisis may entail disaster or recovery for global health sector efforts for HIV and tuberculosis epidemic control. International health agencies should review policies and progress in epidemic control of HIV and tuberculosis, with the aim of identifying synergies: learning mutual lessons for policy development and for scaling up implementation of interventions and identifying ways of joint planning and joint funding of integrated services as part of strengthened health systems.

Although the global financial crisis poses a danger of increased funding gaps for HIV and tuberculosis epidemic control programmes, it also represents an opportunity to re-think established practices and policies, with the aim of delivering better results through greater health system synergies with increased cost-effectiveness. The silver lining in the global economic crisis could be better control of the HIV and tuberculosis epidemics, better overall health system performance and outcomes, and better value for money.

## Summary

• The global financial crisis represents both a danger and an opportunity for global health sector efforts for HIV and tuberculosis epidemic control.

• HIV and tuberculosis share many similarities in terms of their disease burden and the stratagems for epidemic control.

• Despite recent progress, collaboration between HIV and tuberculosis remains inadequate, progress in obtaining synergies has been slow, and results remain far below those needed to achieve the goal of universal access to key interventions.

• International and national health agencies should review policies and progress in epidemic control of HIV and tuberculosis, with the aim of identifying synergies: learning mutual lessons for policy development and for scaling up implementation of interventions and identifying ways of joint planning and joint funding of integrated services as part of strengthened health systems.

• The silver lining in the global economic crisis could be better control of the HIV and tuberculosis epidemics, better overall health system performance and outcomes, and better value for money.

## Competing interests

The author declares that they have no competing interests.

## Authors' contributions

I am the sole author

## Pre-publication history

The pre-publication history for this paper can be accessed here:

http://www.biomedcentral.com/1471-2458/10/394/prepub
